# Ginsenoside Rg-1 prevents elevated cytosolic Ca^2+^ via store-operated Ca^2+^ entry in high-glucose–stimulated vascular endothelial and smooth muscle cells

**DOI:** 10.1186/s12906-022-03647-5

**Published:** 2022-06-22

**Authors:** A Young Han, Su Min Ha, You Kyoung Shin, Geun Hee Seol

**Affiliations:** 1 Department of Basic Nursing Science, College of Nursing, Korea University, 145 Anam-ro, Seongbuk-gu, Seoul, 02841 Republic of Korea; 2 Department of Nursing, College of Life Science and Industry, Sunchon National University, Suncheon, Republic of Korea; 3 BK21 FOUR Program of Transdisciplinary Major in Learning Health Systems, Graduate School, Korea University, Seoul, Republic of Korea

**Keywords:** Ginsenoside Rg-1, Store-operated Ca^2+^ entry, High glucose, Vascular endothelial cells, Vascular smooth muscle cells

## Abstract

**Background:**

Ginsenoside Rg-1 (Rg-1), a triterpenoid saponin abundantly present in *Panax ginseng*, is a type of naturally occurring steroid with known anti-diabetic and anti-inflammatory effects. In this study, we sought to confirm the effects and mechanisms of action of Rg-1 on store-operated Ca^2+^ entry (SOCE) in human vascular endothelial cell line (EA) and murine aortic vascular smooth muscle cell line (MOVAS) cells exposed to high glucose.

**Methods:**

Cytosolic Ca^2+^ concentrations in EA and MOVAS cells were measured by monitoring fluorescence of the ratiometric Ca^2+^-indicator, Fura-2 AM.

**Results:**

High glucose significantly increased Ca^2+^ influx by abnormally activating SOCE in EA and MOVAS cells. Notably, this high glucose-induced increase in SOCE was restored to normal levels in EA and MOVAS cells by Rg-1. Moreover, Rg-1 induced reductions in SOCE in cells exposed to high glucose were significantly inhibited by the plasma membrane Ca^2+^ ATPase (PMCA) blocker lanthanum, the Na^+^/K^+^-ATPase blocker ouabain, or the Na^+^/Ca^2+^ exchanger (NCX) blockers Ni^2+^ and KB-R7943. These observations suggest that the mechanism of action of Rg-1 inhibition of SOCE involves PMCA and Na^+^/K^+^-ATPase, and an increase in Ca^2+^ efflux via NCXs in both EA and MOVAS cells exposed to high glucose.

**Conclusions:**

These findings indicate that Rg-1 may protect vascular endothelial and smooth muscle cells from Ca^2+^ increases following exposure to hyperglycemic conditions.

## Background

Ca^2+^, a second messenger involved in vast array of cellular processes such as metabolic signals, energy production, cell viability, and apoptosis [1]. Ca^2+^ signals are generated by Ca^2+^ influx from the extracellular space via plasma membrane Ca^2+^ channels and through intracellular release from the endoplasmic reticulum (ER)/sarcoplasmic reticulum (SR) via Ca^2+^-release channels [2]. Store-operated Ca^2+^ entry (SOCE) is an important extracellular Ca^2+^-influx mechanism in vascular endothelial and smooth muscle cells [3]. In this mechanism, depletion of ER/SR Ca^2+^ causes stromal interaction molecule 1 (STIM1) to bind to the ER-plasma membrane and signal the Orai channel to trigger Ca^2+^ influx into the cytosol [4]. SOCE is critical to the primary Ca^2+^ signaling pathway of cells and plays an essential role in a wide range of physiological functions, including extracellular excretion, enzyme activity, gene transcription, cell proliferation, and apoptosis [5].

High glucose causes an imbalance in cytosolic Ca^2+^ homeostasis. High glucose upregulates STIM1 and increases Orai1 protein levels, thereby activating SOCE and increasing Ca^2+^ influx [6, 7]. Previous studies have reported that STIM1 and Orai1 proteins are overexpressed in human aortic endothelial cells exposed to high glucose, resulting in increased Ca^2+^ influx through SOCE [8], and have further shown that chronic exposure to high glucose significantly increases apoptosis in human umbilical vein endothelial cells owing to increased hydrogen peroxide production and SOCE-mediated activation of calcineurin [9]. Similar to what is observed in vascular endothelial cells, SOCE and Orai1 protein levels were significantly increased in human aortic smooth muscle cells exposed to high glucose [10].

Ca^2+^ is intimately involved in the regulation of most cellular functions, such that even relatively small disturbances in Ca^2+^ homeostasis can have fatal consequences for cellular function [11]. Disruption of Ca^2+^ homeostasis causes vascular endothelial dysfunction by pathologically activating endothelial nitric oxide synthase [12] and increases the frequency of Ca^2+^ spikes in vascular smooth muscle cells, leading to excessive vasoconstriction [13]. Therefore, an imbalance in cytosolic Ca^2+^ homeostasis induced by high glucose is thought to have crucial effects on vascular health.

**Fig. 1:**
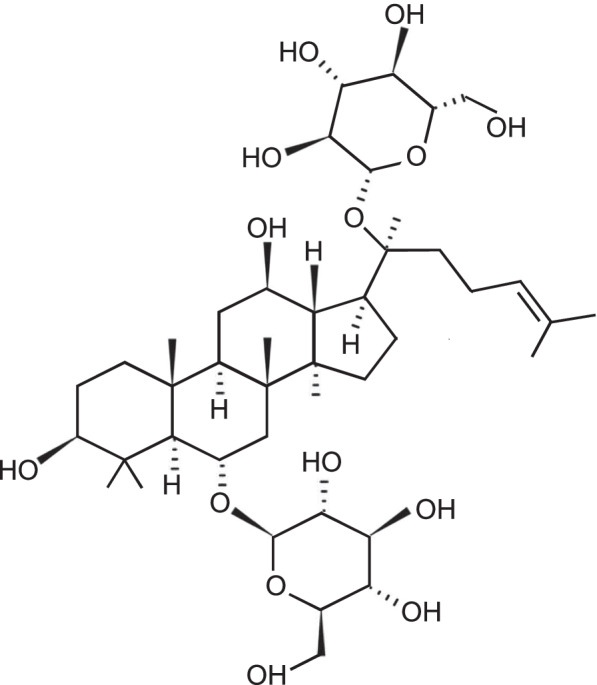
Molecular structure of ginsenoside Rg-1

*Panax ginseng* is widely used in traditional herbal medicine owing to the pharmacological action of its ginsenoside saponins [14]. Among the 150 types of ginsenoside saponins, ginsenosides Rg-1 (Fig. 1), Rb-1 and Rg3 have demonstrated various pharmacological actions on cardiovascular, neuronal, and immune systems [15]. Moreover, ginsenosides are metformin mimetics with anti-diabetes properties [16]; among them, ginsenoside Rg-1 (hereafter, Rg-1) in particular, is known to have an excellent anti-diabetes profile. Rg-1 has also been demonstrated to directly protect pancreatic β-cell function and viability through the PI3K/Akt pathway [17]. Many studies have reported that Rg-1 not only inhibits the deterioration in the viability of cells exposed to high glucose, but also protect against cell damage. However, there are no reports of the effects of Rg-1 on SOCE or the underlying mechanism in vascular cells exposed to high glucose. Accordingly, we herein sought to investigate the preventive effects and related mechanisms of Rg-1 on SOCE in a human endothelial cell line (hereafter, EA) and a murine aortic vascular smooth muscle cell line (MOVAS) under high glucose.

## Methods

### Materials

Rg-1, Rb-1, glucose, lanthanum (La^3+^), ouabain, nickel (Ni^2+^) and KB-R7943 were purchased from Sigma-Aldrich (St. Louis, MO, USA). MTT (3-[4,5-dimethylthiazol-2-yl]-2,5 diphenyl tetrazolium bromide) solution was from Amresco (Solon, OH, USA), and Fura-2 AM was supplied by Molecular Probes (Eugene, OR, USA).

### Cell culture and hyperglycemia induction

EA cells, originally isolated from human umbilical veins, were purchased from the American Type Culture Collection (Manassas, VA, USA). MOVAS cells, isolated from aortic smooth muscle cells of C57BL6 mice, were purchased from Charles River Laboratories (Wilmington, MA, USA). EA and MOVAS cells were maintained in Dulbecco’s modified Eagle’s medium supplemented with 10% fetal bovine serum, 1% MEM non-essential amino acids, penicillin, and streptomycin. For culture under high-glucose conditions, cells were exposed to medium containing 30 mM glucose for 48 hours [18].

### Cell viability assay

The optimal concentration of Rg-1 was determined by first confirming cell viability using the MTT technique, according to the procedure described in a previous study [19]. Briefly, cells were seeded in 96-well plates at 1 × 10^4^ cells per well in 200-μl aliquots. Cells were incubated with 0 to 50 μM Rg-1 at 37 °C for 48 hours. Each well was then washed with phosphate-buffered saline, followed by addition of a pure-grade MTT (5 mg/ml) solution to each well. After incubation for 3 hours, 100 ml dimethyl sulfoxide (DMSO) was added to each well to dissolve formazan crystals generated by reduction of MTT by metabolically active cells. Plates were incubated for an additional 3 hours in the dark, and the optical density of each well was measured at 540 nm using a plate reader (Spectrostar Nano; BMG LABTECH, Ortenberg, Germany).

### Cytosolic Ca^2+^ measurement

EA and MOVAS cells were exposed to high glucose with or without Rg-1 for 48 hours. Cytosolic Ca^2+^ was then measured using Fura-2 AM, a membrane-permeable, fluorescent ratiometric Ca^2+^-binding dye [20]. Approximately 1 × 10^6^/ml cells were incubated with 2.5 μM Fura-2 AM in the dark for 30 minutes at room temperature. Dye remaining in the extracellular fluid was removed by exchanging the supernatant twice. Cells were placed in a quartz cuvette, and fluorescence at 510 nm was measured using a fluorescence spectrometer (Photon Technology Instruments) with alternating wavelengths of 340 nm and 380 nm with a chopper wheel (50 Hz). Cytosolic Ca^2+^ was expressed as the ratio of fluorescence at 340 nm to fluorescence at 380 nm (F340/F380).

### Data analysis and statistics

All statistical analyses were performed using SPSS Statistics software version 26 (IBM, IL, USA). Data are expressed as means ± standard error of the mean (SEM). Differences among groups were analyzed by analysis of variance and Fisher’s Least Significant Difference post-hoc test. *p*-values < 0.05 were considered statistically significant.

## Results

### Effects of ginsenosides Rg-1 and Rb-1 on high-glucose–exposed EA and MOVAS cells

**Fig. 2:**
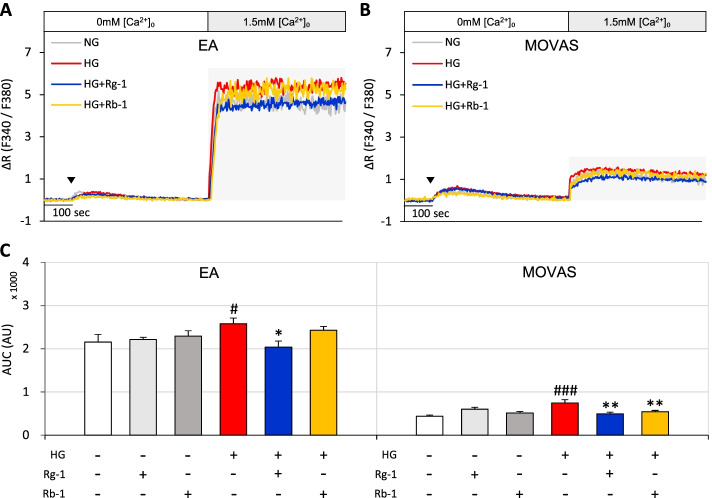
Effects of Rg-1 on SOCE in EA and MOVAS cells exposed to high glucose compared with those of Rb-1. **A** Representative traces showing the effects of normal glucose, high glucose, and high glucose with 10 μM Rg-1 or 10 μM Rb-1, on SOCE in EA cells. **B** Representative traces showing the effects of normal glucose, high glucose, and high glucose with 10 μM Rg-1 or 10 μM Rb-1, on SOCE in MOVAS cells. **C** Summary data showing area under the curve (AUC) values for 500 seconds for EA and MOVAS cells. Data are means ± SEM (*n* = 6–14; ^#^*p* < 0.05, ^###^*p* < 0.001 compared with normal glucose; ^*^p < 0.05, ^**^p < 0.01 compared with high glucose). The symbol (▼) indicates the time point at which 30 μM BHQ was applied

First, we compared the effects of Rg-1 and Rb-1 on SOCE in EA and MOVAS cells exposed to high glucose. In the normal glucose group, the effects of Rg-1 and Rb-1 on SOCE did not significantly differ between EA and MOVAS cell. However, the SOCE-inhibitory effects of Rg-1 and Rb-1 were different in the high-glucose group, with Rg-1 significantly reducing SOCE in both EA and MOVAS cells and Rb-1 significantly decreasing SOCE only in MOVAS cells (Fig. 2A-C). These findings confirmed that, of the ginsenosides evaluated, Rg-1 had a greater effect than Rb-1 on Ca^2+^ homeostasis in EA and MOVAS cells exposed to high glucose.

### Optimal Rg-1 concentration inhibiting SOCE in high-glucose–exposed EA and MOVAS cells

**Fig. 3:**
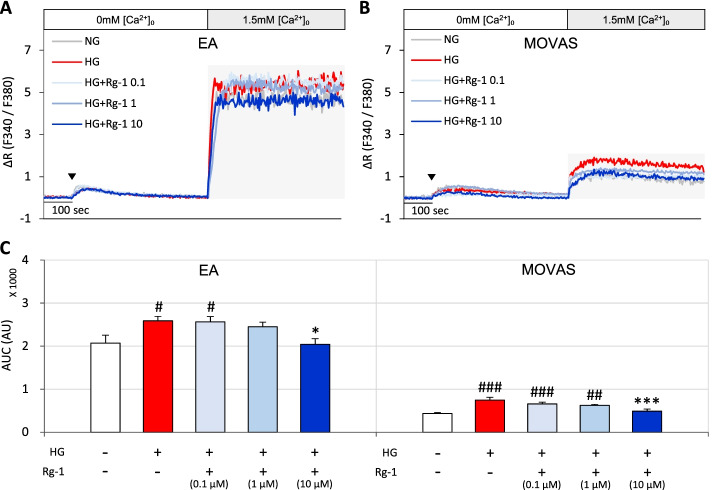
Effects of various concentrations of Rg-1 on SOCE in EA and MOVAS cells exposed to high glucose. **A** & **B** Representative traces showing effects of normal glucose, high glucose, and high glucose plus 0.1, 1, or 10 μM Rg-1 on SOCE in **(A)** EA cells and (**B)** MOVAS cells. **C** Summary data showing area under the curve (AUC) values for 500 seconds for EA and MOVAS cells. The symbol (▼) indicates the time point at which 30 μM BHQ was applied. Data are means ± SEM (*n* = 6–11; ^#^*p* < 0.05, ^##^*p* < 0.01, ^###^*p* < 0.001 compared with normal glucose; ^*^p < 0.05, ^***^p < 0.001 compared with high glucose)

The optimal Rg-1 concentration inhibiting SOCE was determined by the MTT method. Incubation of EA and MOVAS cells with 0, 0.01, 0.1, 1, 10, 20 or 50 μM Rg-1 showed that Rg-1 at concentrations above 20 μM significantly reduced cell viability compared with controls (data not shown). In addition, the effect of Rg-1 on the degree of SOCE inhibition was assessed by incubating EA and MOVAS cells exposed to high glucose with 0.1, 1, or 10 μM Rg-1. In both cell types, 10 μM Rg-1 concentration significantly reduced the high glucose-induced increase in SOCE, restoring normal glucose levels, whereas neither 0.1 nor 1 μM Rg-1 had any effect (Fig. 3A-C). Thus, Rg-1 was used at a concentration of 10 μM in subsequent experiments.

### Mechanisms of SOCE inhibition by ginsenoside Rg-1 on high-glucose–exposed EA and MOVAS cells

The mechanism of SOCE inhibition by Rg-1 was analyzed by comparing its effect with the effects of the plasma membrane Ca^2+^ ATPase (PMCA) blocker La^3+^; the Na^+^/K^+^ ATPase blocker ouabain; and the Na^+^/Ca^2+^ exchanger (NCX) blockers, Ni^2+^ and KB-R7943. Specifically, KB-R7943 is an inhibitor of the reverse mode of Na^+^/Ca2^+^ exchanger.

**Fig. 4:**
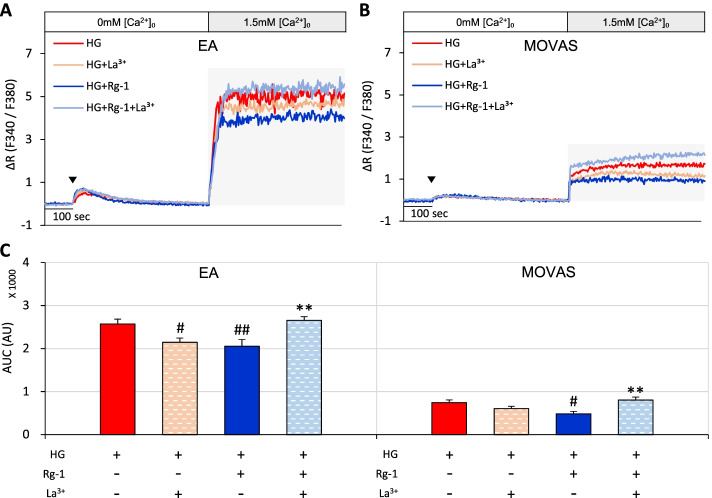
Effects of La^3+^ on Rg-1–induced increases in SOCE in EA and MOVAS cells exposed to high glucose. **A** & **B** Representative traces showing the effects of high glucose, high glucose plus 125 μM La^3+^, high glucose plus 10 μM Rg-1, and high glucose plus 10 μM Rg-1 and 125 μM La^3+^ on SOCE in (**A)** EA and (**B)** MOVAS cells. **C** Summary data showing area under the curve (AUC) values for 500 seconds for EA and MOVAS cells. The symbol (▼) indicates the time point at which 30 μM BHQ was applied. Data are means ± SEM (*n* = 7–11; ^#^*p* < 0.05, ^##^*p* < 0.01 compared with high glucose; ^**^p < 0.01 compared with high glucose plus Rg-1)

SOCE was more substantially inhibited in cells pretreated with La^3+^ plus Rg-1 compared with cells pretreated with La^3+^ alone. La^3+^ alone significantly decreased SOCE in high-glucose–exposed EA cells, but not MOVAS cells. Notably, La^3+^ plus Rg-1 resulted in a significantly greater increase in SOCE than Rg-1 alone in both EA and MOVAS cells exposed to high glucose (Fig. 4A-C). These results suggest that the mechanism by which Rg-1 decreases SOCE in EA and MOVAS cells exposed to high glucose may be associated with the PMCA pathway.

**Fig. 5:**
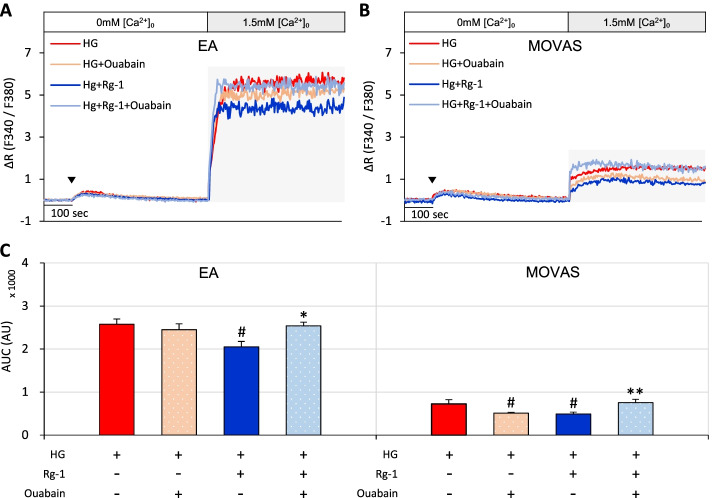
Effects of ouabain on Rg-1–induced increases in SOCE in EA and MOVAS cells exposed to high glucose. **A** & **B** Representative traces showing the effects of high glucose, high glucose plus 10 nM ouabain, high glucose plus 10 μM Rg-1, and high glucose plus 10 μM Rg-1 and 10 nM ouabain on SOCE in (**A)** EA and (**B)** MOVAS cells. **C** Summary data showing area under the curve (AUC) values for 500 seconds for EA and MOVAS cells. The symbol (▼) indicates the time point at which 30 μM BHQ was applied. Data are means ± SEM (*n* = 6–11; ^#^*p* < 0.05 compared with high glucose; ^*^p < 0.05, ^**^p < 0.01 compared with high glucose plus Rg-1)

To determine whether the SOCE inhibitory effect of Rg-1 was related to Na^+^/K^+^ channels, EA and MOVAS cells exposed to high glucose were pretreated with Rg-1, the Na^+^/K^+^ ATPase blocker ouabain, or both. Treatment of cells exposed to high glucose with ouabain or Rg-1 alone reduced SOCE compared with controls. Compared with Rg-1 alone, however, Rg-1 plus ouabain significantly increased SOCE (Fig. 5A-C). This suggests that Rg-1 increases Ca^2+^ efflux in EA and MOVAS cells exposed to high glucose via the Na^+^/K^+^ ATPase pathway.

**Fig. 6:**
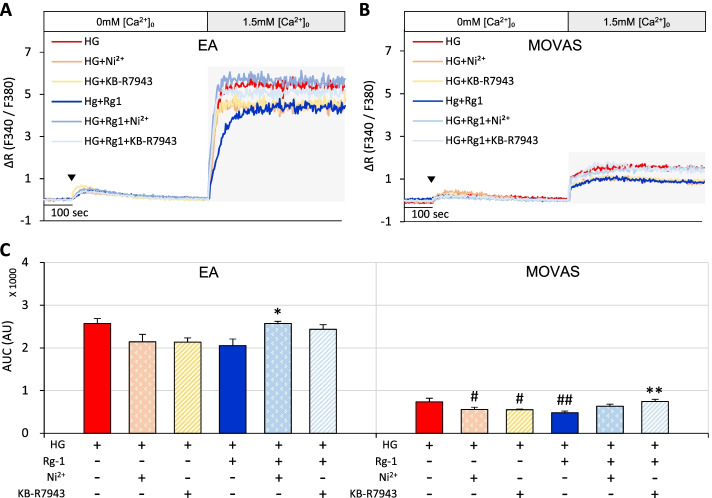
Effects of Ni^2+^ and KB-R7943 on Rg-1–induced increases in SOCE in EA and MOVAS cells exposed to high glucose. **A** & **B** Representative traces showing the effects of high glucose, high glucose plus 100 nM Ni^2+^, high glucose plus 10 μM KB-R7943, high glucose plus 10 μM Rg-1, high glucose plus 10 μM Rg-1 and 100 nM Ni^2+^, and high glucose plus 10 μM Rg-1 and 10 μM KB-R7943 on SOCE in (**A)** EA and (**B)** MOVAS cells. **C** Summary data showing area under the curve (AUC) values for 500 seconds for EA and MOVAS cells. The symbol (▼) indicates the time point at which 30 μM BHQ was applied. Data are means ± SEM (*n* = 6–12; ^#^*p* < 0.05, ^##^*p* < 0.01 compared with high glucose; ^*^p < 0.05, ^**^p < 0.01 compared with high glucose plus Rg-1)

The NCX pathway has been reported to be effective in coping with increased intracellular Ca^2+^ in pancreatic cells [21]. Therefore, we assessed whether the Rg-1-induced reduction in SOCE was related to the NCX pathway. Treatment of EA and MOVAS cells with Ni^2+^ or KB-R7943 inhibited the high glucose-induced increase in SOCE to a level similar to that of Rg-1. However, SOCE was more substantially increased in cells pretreated with Rg-1 plus Ni^2+^ or Rg-1 plus KB-R7943 than in cells pretreated with Ni^2+^ or KB-R7943 alone, respectively (Fig. 6A-C).

Collectively, these findings indicate that high glucose significantly increases Ca^2+^ influx by abnormally activating SOCE in EA and MOVAS cells, and that Rg-1 reverses SOCE by increasing Ca^2+^ efflux through PMCA, Na^+^/K^+^ ATPase, and NCX.

## Discussion

**Fig. 7:**
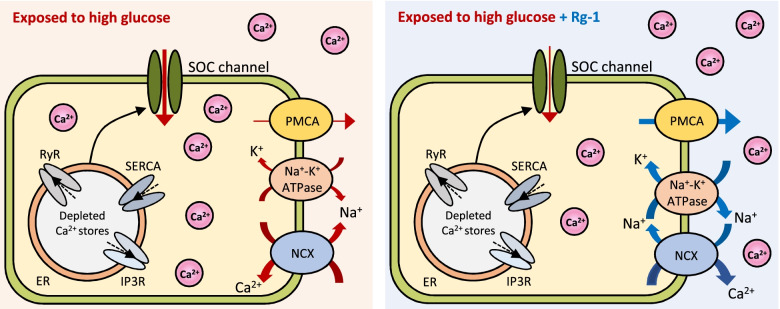
Action mechanisms of Rg-1 on SOCE in EA and MOVAS cells exposed to high glucose. The red and blue colors indicate changes in the regulation of intracellular Ca^2+^ levels by exposure to high glucose or Rg-1 treatment, respectively. The thicknesses of the arrows reflect the amount of Ca^2+^ influx or efflux

In vascular endothelial and smooth muscle cells, the SOCE pathway, activated by depletion of intracellular Ca^2+^ stores, plays an important role in regulating intracellular functions [3]. High glucose exposure promotes enhanced permeability and proliferation of human coronary artery endothelial cells through increases in SOCE via Orai-mediated, Ca^2+^ release-activated Ca^2+^ channels [22]. Notably in this context, it has been reported that diabetic hyperglycemia increases the expression of Orai1 and SOCE in vascular smooth muscle cells [10]. Our findings demonstrated that exposure to high glucose resulted in a significant increase in SOCE in EA and MOVAS cells; this effect was inhibited by Rg-1 treatment, which restored Ca^2+^ to levels found under normal glucose conditions; this Rg-1-associated inhibition of SOCE in EA and MOVAS cells exposed to high glucose might be related to the PMCA, Na^+/^K^+^ ATPase, and NCX pathways (Fig. 7). Notably, Rg-1 did not have a significant SOCE-inhibitory effect under normoglycemic conditions, but only exhibited this action under hyperglycemic conditions. In a similar vein, a study investigating the blood pressure effects of *Codonopsis lanceolata*, a natural plant rich in triterpenoid saponins, showed no effect on blood pressure in the normotensive group, but exerted a significant blood pressure-lowering effect in the hypertensive group [23].

In this study, treatment with Ca^2+^ antagonists such as La^3+^, ouabain, Ni^2+^, and KB-R7943 reduced SOCE in cells exposed to high glucose concentrations. However, incubation of cells with both Rg-1 and Ca^2+^ antagonists resulted in a rapid increase in SOCE. Increased intracellular Ca^2+^ is returned to the extracellular compartment by the action of the PMCA. This was shown, for example, by treatment of cells with 125 μM La^3+^, which blocked PMCA [24]. Treatment of pancreatic beta cells with 22.2 mM glucose resulted in significantly greater reductions in PMCA1 and PMCA2 mRNAs than treatment with 2.8 mM glucose [25]. These findings, showing that PMCA activity was inhibited under high glucose conditions, suggest that the inhibition of PMCA may have affected the increase in SOCE under high glucose than normal glucose conditions. In addition, the inhibition of SOCE by a relatively high concentration of La^3+^ under high glucose conditions may be due to its blocking of non-selective cation channels. Conversely, the increase in SOCE induced by treatment with both La^3+^ and Rg-1 under high glucose conditions may be related to the Rg-1-induced increase of Ca^2+^ efflux through PMCA, which inhibits SOCE under high glucose conditions.

Exposure of pancreatic β-cells to glucose leads to conversion of a PMCA-based, low-efficiency Ca^2+^ efflux mechanism to an NCX-based, high-efficiency system that is better able to cope with glucose-induced Ca^2+^ influx [21]. As the glycemic load in pancreatic β-cells increases, it induces a concentration-dependent decrease in PMCA activity, while significantly increasing NCX activity [25]. NCX is a reversible transporter of Ca^2+^ across plasma membranes. Exposure to abnormally high glucose concentrations has been reported to increase NCX activity and contribute to diabetic microvascular complications [26, 27]. This increase in NCX activity was inhibited by KB-R7943, suggesting that hyperglycemia-induced NCX activity is strongly associated with the NCX reverse mode [28]. In the present study, Rg-1 treatment inhibited the abnormally increased SOCE induced by high glucose concentrations, suggesting that Rg-1 may inhibit Ca^2+^ influx through the NCX reverse mode. In contrast, treatment with both Rg-1 and KB-R7943 under high glucose conditions resulted in a greater increase in SOCE than high glucose alone. Because pretreatment with KB-R7943 was found to increase the intracellular basal sodium concentration in cardiomyocytes of diabetic rats [27], the increased potential difference may depolarize cells and stimulate voltage-dependent calcium channels. When Ca^2+^ influx is excessively disturbed, various ion channels, such as NCXs or voltage-operated Ca^2+^ channels, play an important role in generating the Ca^2+^ influx underlying oscillations [29–31]. Future studies needed to assess the effects of Rg-1 on the expression of Ca^2+^ channel proteins, especially the expression of proteins that regulate SOCE, such as Orai1, Orai2, Orai3, STIM1, and STIM2.

Hyperglycemia-induced metabolic dysfunctions, such as enhanced formation of advanced glycation end products (AGEs) and increased production of reactive oxygen species, have been suggested as factors for developing diabetic vascular complications [32]. In mouse mesangial cells, high glucose enhanced the formation of methylglyoxal (the major precursor of AGEs) and the level of 8-OHdG, suggesting increased oxidative stress [33]. In line with these reports, catalase treatment of platelets from patients with type 2 diabetes mellitus increased the PMCA tyrosine phosphorylation induced by thapsigargin plus ionomycin, suggesting that oxidative stress is involved in the reduced platelet PMCA activity seen in diabetic patients [34]. High glucose also significantly increased NCX activity and malondialdehyde production in human umbilical vascular endothelial cells, and this effect was abolished by KB-R7943-mediated inhibition of the reverse mode of NCX, indicating that increased reverse-mode NCX activity plays an important role in high glucose-induced endothelial dysfunction [28]. Rg-1 has demonstrated several pharmacological actions including antioxidant effects [35]. Rg-1 reduces reactive oxygen species (ROS) by modulating Nrf2 (nuclear factor-erythrocyte 2-related factor 2) and NLRP3 (nucleotide-binding oligomerization domain-like receptor protein 3) signaling pathways [36]. Several studies have shown that hyperglycemia induces lipid peroxidation, nitrite production, and intracellular ROS formation [37, 38]. In the current study, the reduction in SOCE is probably related to the antioxidant activity of Rg-1.

In vascular endothelial cells, Rg-1 produced a more prominent SOCE-reducing effect than Rb-1. Although few studies have investigated the regulation of intracellular Ca^2+^ in vascular cells by Rb-1, one study in rat cortical synaptosomes showed that it decreased intracellular Ca^2+^ by increasing Na^+^/K^+^-ATPase and Ca^2+^/Mg^2+^-ATPase activity [39]. In addition, Rb-1 protects cardiomyocytes by reducing the high-Ca^2+^–induced delayed afterdepolarizations of cardiomyocytes. On the other hand, Rg-1 has been reported to have Ca^2+^-regulating effects in various cell types. In an ischemia-reperfused hippocampal cell model, it was shown to have neuroprotective effects by blocking excess Ca^2+^ influx and decreasing neuronal nitric oxide synthase activity [40]. In myocardial cells, it has been shown that, Rg-1 restores cellular homeostasis by reducing ROS and inhibiting Ca^2+^ influx [41]. In neurons, Rg-1 shows a cell-protective effect through a reduction in intracellular free Ca^2+^ [42] and exerts a Ca^2+^ homeostasis-maintenance effect by reducing ROS and oxidative stress and protecting against neuroinflammation [43]. In lymphocytes, Rg-1 has been shown to inhibit Ca^2+^ influx attributable to H_2_O_2_-induced damage and significantly reduce apoptosis and lymphocyte damage caused by oxidative stress, indicating protective effects on immune cells [44]. Therefore, Rg-1 exerts cytoprotective effects under pathological conditions by maintaining Ca^2+^ homeostasis. However, further studies are needed to show the different mechanism of Rg-1 in different cell types such as endothelial cell and smooth muscle cells.

## Conclusions

Present study demonstrated that treatment with Rg-1 may be a new approach to protecting vascular endothelial and smooth muscle cells in patients with hyperglycemia through maintenance of Ca^2+^ homeostasis.

## Data Availability

All data generated or analyzed during this study are included in this published article.

## Abbreviations

AGE: Advanced glycation end product
AUC: Area under the curve
DMSO: Dimethyl sulfoxide
EA: Human endothelial cell line
ER: Endoplasmic reticulum
MOVAS: Murine aortic vascular smooth muscle cell line
NCX: Na^+^/Ca^2+^ exchanger
PMCA: Plasma membrane Ca^2+^ ATPase
SEM: Standard error of the mean
SOCE: Store-operated Ca^2+^ entry
SR: Sarcoplasmic reticulum
STIM: Stromal interaction molecule
ROS: Reactive oxygen species
